# Role of human milk oligosaccharides in Group B Streptococcus colonisation

**DOI:** 10.1038/cti.2016.43

**Published:** 2016-08-26

**Authors:** Nicholas J Andreas, Asmaa Al-Khalidi, Mustapha Jaiteh, Edward Clarke, Matthew J Hyde, Neena Modi, Elaine Holmes, Beate Kampmann, Kirsty Mehring Le Doare

**Affiliations:** 1 Department of Paediatrics, Centre for International Child Health, Imperial College London, St Mary's Hospital, London, UK; 2 Department of Medicine, Section of Neonatal Medicine, Imperial College London, London, UK; 3 The Centre for Digestive and Gut Health, Imperial College London, London, UK; 4 Section of Computational and Systems Medicine, Faculty of Medicine, Imperial College London, London, UK; 5 MRC Unit-The Gambia, Vaccines and Immunity Theme, Fajara, The Gambia; 6 Wellcome Trust Centre for Global Health Research, London, UK

## Abstract

Group B Streptococcus (GBS) infection is a major cause of morbidity and mortality in infants. The major risk factor for GBS disease is maternal and subsequent infant colonisation. It is unknown whether human milk oligosaccharides (HMOs) protect against GBS colonisation. HMO production is genetically determined and linked to the Lewis antigen system. We aimed to investigate the association between HMOs and infant GBS colonisation between birth and postnatal day 90. Rectovaginal swabs were collected at delivery, as well as colostrum/breast milk, infant nasopharyngeal and rectal swabs at birth, 6 days and days 60–89 postpartum from 183 Gambian mother/infant pairs. GBS colonisation and serotypes were determined using culture and PCR. ^1^H nuclear magnetic resonance spectroscopy was used to characterise the mother's Lewis status and HMO profile in breast milk. Mothers who were Lewis-positive were significantly less likely to be colonised by GBS (*X*^2^=12.50, *P*<0.001). Infants of Lewis-positive mothers were less likely GBS colonised at birth (*X*^2^=4.88 *P*=0.03) and more likely to clear colonisation between birth and days 60–89 than infants born to Lewis-negative women (*P*=0.05). There was no association between Secretor status and GBS colonisation. *In vitro* work revealed that lacto-*N*-difucohexaose I (LNDFHI) correlated with a reduction in the growth of GBS. Our results suggest that HMO such as LNDFHI may be a useful adjunct in reducing maternal and infant colonisation and hence invasive GBS disease. Secretor status offers utility as a stratification variable in GBS clinical trials.

*Streptococcus agalacticae* (Group B Streptococcus (GBS)) is a Gram-positive bacterium that colonises the maternal gastrointestinal tract and vagina.^1^ GBS is the leading cause of infection in the first 3 months of life in the United Kingdom^2^ and United States of America^3^ and is increasingly described as a major cause of infection in Sub-Saharan Africa.^4, 5, 6^ GBS is vertically transmitted to approximately 50% of neonates born to colonised mothers and causes pneumonia, sepsis and/or meningitis in approximately 1–2% of these infants in the first 3 postnatal months.^7^ Maternal and subsequent infant colonisation precedes invasive disease.^8^

In order to resist infection, the neonate is initially reliant on maternal protection, primarily via transplacentally derived immunoglobulin G (IgG). However, IgG mainly provides protection once a pathogen has already entered the blood stream.^9^ In addition to maternally derived IgG in blood, breast-fed infants obtain protection against various pathogens through many bioactive factors in breast milk.^10^ Breast feeding is important for neonatal health and decreases infant susceptibility to gastrointestinal and respiratory tract infections, botulism and necrotising enterocolitis, and reduces mortality.^9, 11, 12^

Protection by breast milk occurs primarily at the mucosal surface from factors including secretory IgA and human milk oligosaccharides (HMOs).^9, 13^ HMOs are soluble complex carbohydrates. The biosynthesis of HMO structures is known to depend on maternal genotype, including the genes that determine the Lewis blood group antigen, which regulates the expression and activity of several different glycosyltransferase enzymes in the mammary tissue. These determine different HMO profiles and their concentrations in breast milk.^14, 15, 16, 17^ For example, a fucosyltransferase enzyme, FUT3, dependent on Lewis gene expression, attaches fucose in an α1–3 or α1–4 linkage, elongating the HMO chain and producing different types of HMOs, depending on the links between the monosaccharides and their stereochemical configuration.^17^ Similarly, an additional group of HMOs, including 2′-fucosyllactose (2′-FL), is only synthesised if the woman has an active copy of the Secretor gene (FUT2) and therefore expresses the α1–2-fucosylatransferease enzyme, responsible for synthesising 2′-FL, as well as other structurally similar HMOs.^15^

Once ingested by the infant, multiple functions have been attributed to HMOs, including the ability to inhibit the adherence of pathogens to the intestinal epithelium. By acting as decoy receptors and thereby preventing pathogen attachment to host cells, HMOs inhibit invasion and subsequent infection.^18^ For example, *Campylobacter jejuni* is less likely to infect infants of mothers whose breast milk contains high concentrations of the HMO 2′-FL.^13^

The Lewis blood group type of children in Burkina Faso and Nicaragua is associated with susceptibility to and incidence of rotavirus infection owing to host phenotype and pathogen genotype. These observations provide an explanation for the reduced efficacy of the live oral rotavirus vaccine in Africa.^19^ Similarly, an observational study in the United States found severe rotavirus gastroenteritis to be absent in non-secretor children, providing important evidence into the epidemiology of this infection and the likely efficacy of vaccination in different populations.^20^

Several *in vivo* studies have also identified the ability of HMOs to reduce *Streptococcus pneumoniae* colonisation of the oropharynx.^21^

HMOs also provide a source of energy for the non-pathogenic intestinal microbiota,^22^ thus preventing infection by allowing the microbiota to outcompete potential pathogenic organisms.^23, 24^

Because of these beneficial effects, it has been proposed that HMOs could be used therapeutically, for example, as an adjunct to standard antibiotics.^25, 26^

HMO research to date has primarily focused on the antiadhesive effects against gut viruses and bacteria *in vitro*. Work of other investigators indicates that GBS is unable to proliferate in the presence of specific HMO *in vitro*,^26^ with certain non-sialylated HMOs identified as possessing a bacteriostatic effect against GBS. Further *in vitro* investigation revealed that GBS uses a glycosyltransferase, which incorporates HMOs into the cell membrane, preventing bacterial proliferation. This mechanism of action is similar to various classes of antibiotic. Furthermore, a GBS mutant lacking the gene encoding for this glycosyltransferase enzyme was found to be non-susceptible to the bacteriostatic effects of HMOs.^26^

We used ^1^H nuclear magnetic resonance (NMR) spectroscopy to test the hypothesis that the type and quantity of HMOs in breast milk influences GBS colonisation status in mothers and their breast-fed infants. Furthermore, we used an *in vitro* challenge model to identify which HMOs were associated with reduction in GBS growth.

## Results

### Metabolic phenotyping of breast milk HMOs

**Figure 1:**
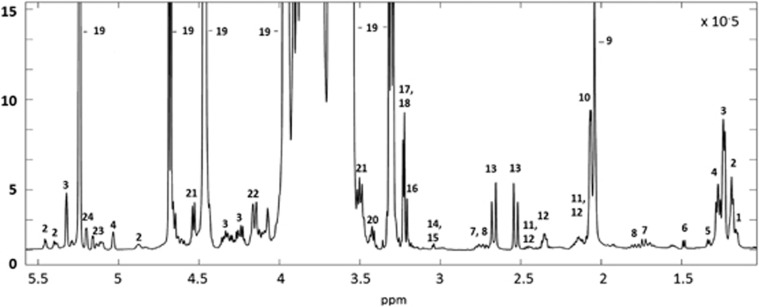
Representative ^1^H NMR spectra of the aqueous fraction of breast milk. 1, LNDFHII; 2, α1–3 fucosyllated oligosaccharides; 3, α1–2 fucosyllated oligosaccharides; 4, α1–4 fucosyllated oligosaccharides; 5, lactate; 6, Leucine/isoleucine; 7, 6′-sialyllactose; 8, 3′-sialyllactose; 9 and 10, *N*-acetylglucosamine containing oligosaccharides; 11, glutamine; 12, glutamate; 13, citrate; 14, creatine; 15, creatinine; 16, choline; 17, phosphocholine; 18, glycerophosphocholine; 19, lactose; 20, Taurine; 21, glucose/glucose containing oligosaccharides; 22, oligosaccharides containing GlcNAc(β1–6) linkage; 23, LNFPIII and branched chain oligosaccharides; 24, LNDFHI and branched chain oligosaccharides. Adapted with permission from Erney *et al.*^29^

**Figure 2:**
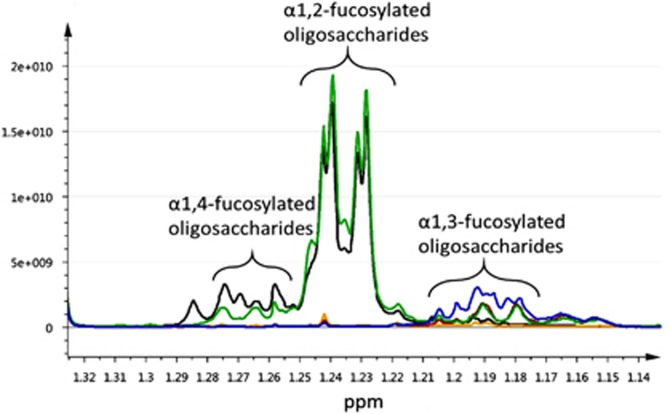
Comparison of spectra of mothers producing different profiles of HMOs. Spectra from mothers in blue, orange and red are non-Secretors, as these spectra do not contain signals corresponding to 2′-FL between *δ* 1.22 and 1.25, while the mothers in green and black are classified as Secretors.

The principal component analysis score plots indicated milk samples were dominated by variance mainly arising from the different fucosylated HMOs, in particular 2′-FL, 3′-fucosyllactose (3′-FL), lacto-*N*-difucohexaose I (LNDFHI), lacto-*N*-difucohexaose II (LNDFHII), lacto-*N*-fucopentaose I, lacto-*N*-fucopentaose III and lactodifucotetraose. Fucosylated HMOs were present in different abundances in the breast milk of different mothers and are indicated in the spectra provided in Figures 1 and 2. Statistical total correlation spectroscopic plots provided more detailed structural definition for each of the HMO (Supplementary Figure S1).

In the colostrum samples (*n*=109), 70% of mothers were identified as Secretors and 30% were identified as non-Secretors. Similarly, 68% of mothers were identified as Lewis positive and 32% were identified as Lewis negative. Non-Secretor mothers appeared to compensate for not producing 2′-fucosylated oligosaccharides by producing an increased quantity of 3′-fucosylactose. Colostrum sample composition was not associated with maternal ethnicity, weight, age, gravida, infant sex or weight at birth or 3 months postpartum, as determined by orthogonal partial least squares (OPLS).

**Figure 3:**
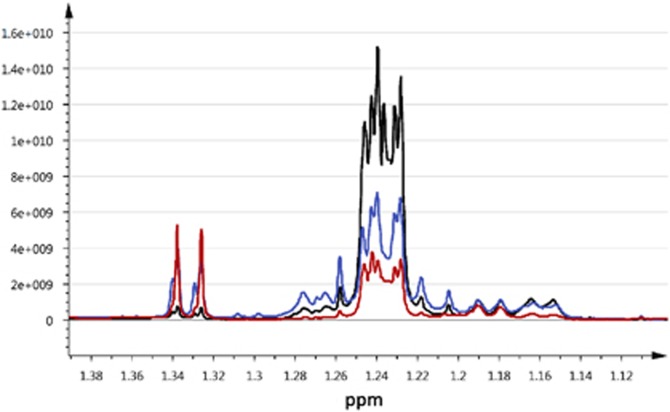
Three overlaid ^1^H NMR spectra from the aqueous fraction of breast milk originating from the same mother collected at 1 day postpartum (black), 6 days postpartum (blue) and 3 months postpartum (red).

Comparing the spectral region containing fucosylated HMOs longitudinally, the HMO profiles remained the same between time points. However, colostrum samples had higher quantities of HMOs in comparison to breast milk (Figure 3).

### Association between HMO profiles and GBS colonisation

We observed a significant negative association between maternal Lewis-positive (Le+) phenotype and maternal GBS colonisation at delivery and for infant GBS colonisation at birth (Table 1). However, this association was not observed for infants at days 60–89, possibly owing to the low numbers of infants colonised at this time point (*n*=19; Table 1).

In contrast, there was no statistically significant difference between maternal or infant GBS colonisation at birth or at days 60–89 between Secretor (Se+) and non-Secretor (Se−) mothers (Table 2).

When combining mothers into milk groups dependent on their Le/Se status, mothers in milk group 3 (Se+/Le−) were more likely to be GBS colonised than any other milk group (*X*^2^=16.57, *P*<0.001, Tables 3 and 4). Infants of mothers in milk group 3 were also more likely to be colonised at birth (Tables 3 and 4).

### Specific HMO types and GBS colonisation in infants and in breast milk

We observed a negative correlation between the relative concentration of 3′-FL and infant GBS colonisation (colony-forming units (CFU) ml^−1^) at birth, (*n*=27, *R*=−0.54, *P*=0.004). There was also a positive correlation between the relative concentration of 2′-fucosyllated oligosaccharides (associated with Se positivity) and infant GBS colonisation (CFU ml^−1^) at birth (*n*=27, *R*=0.45, *P*=0.02).

A similar negative correlation was observed for the concentration of GBS in breast milk and relative concentration of 3′-FL (*n*=10, *R*=−0.66, *P*=0.04). Likewise, 2′-fucosyllated oligosaccharides and GBS abundance in breast milk correlated positively, nearing significance (*n*=10, *R*=0.59, *P*=0.07; Supplementary Tables S1–S3; Supplementary Figures S5 and S6).

Clearance of colonisation between birth and day 6 or between birth and days 60–89 was associated with peaks at 1.29 (F=1.29, *P*=0.12), 5.03 (F=2.62, *P*=0.05), *δ* 5.16 (F=2.09, *P*=0.10), all corresponding to the HMO LNDFHI, which is associated with the Lewis antigen group and only produced by mothers who are both Le+/Se+.

### HMOs and GBS growth in vitro

Presence of LNDFHI and other similar branched HMOs in breast milk were associated with a 50% reduction in GBS growth *in vitro* (*X*^2^=2.05, *P*=0.048). Table 5 displays the Pearson correlation coefficients between peak heights and difference in CFU ml^−1^ over 24 h.

These breast milk originated from women who were both GBS colonised and uncolonised (12 GBS-colonised, 28 GBS-uncolonised at delivery).

## Discussion

Our study findings suggest that Lewis phenotype and its related HMOs in breast milk are strongly associated with inhibition of GBS colonisation in the mother and a reduced risk of transmission to the infant. In addition, we demonstrate that clearance of colonisation in infants is associated with certain HMOs.

We demonstrated a dose-dependent effect on GBS growth *in vivo* and *in vitro* with certain HMO structures associated with Lewis gene activity, primarily LNDFHI and other similar branched HMOs produced only by Le+/Se+ mothers. Furthermore, the HMO 3′-FL was inversely correlated with the abundance of GBS in both infants and breast milk. Our *in vitro* results also suggest a bacteriostatic role for these HMOs against GBS that may be clinically meaningful.

Taken together, our results indicate that LNDFHI and other similar branched HMOs are able to inhibit the growth of GBS. Similar results have been reported in a recent study by Bode *et al.*,^26^ although the details of the specific HMOs involved are not mentioned. Our results indicate a possible role for specific HMOs in the prevention and clearance of maternal GBS colonisation during pregnancy. Once additional studies have been undertaken to validate these results, and definitively identify the HMO(s) involved in protection against GBS disease, a clinical application could be to use HMOs as a potential adjuvant to antibiotics for the treatment of GBS colonisation.

In the clinical context, it may be possible to supplement Lewis-negative mothers with synthetic LNDFHI and other similar branched HMOs during pregnancy and lactation. This supplementation could ‘convert' the mother's Lewis group, in an attempt to reduce the incidence of maternal GBS colonisation, and hence reduce the vertical transmission to their neonates. This supplementation has recently become feasible owing to advances in bioengineering, allowing for various HMOs to be synthesised using whole-cell biocatalysis.^27^

Likewise, these HMOs, particularly LNDFHI and 3′-FL, could be provided to infants of non-Lewis positive mothers colonised with GBS, in an attempt to reduce the likelihood of the infant becoming colonised with GBS.

The percentage of Secretor mothers found in the present study closely reflects the results of a previous study conducted in The Gambia, which reported 73% of mothers as Secretors.^28^ There is considerable variation in HMO type and abundance globally.^29^ The higher proportion of Lewis-positive mothers and lower proportion of Secretor mothers in Asia might account for differences observed in GBS colonisation here, although this is speculative at present.

Our study has several limitations. First, we were unable to quantify exact HMO concentrations owing to the binding of the TSP standard to proteins, which remained in the milk, affecting the TSP concentration and therefore the reference value. To account for this, we used the intensity of the HMO peaks in the spectra, which are directly related to the concentration of these molecules. Second, a further difficulty was the extensive overlap in the ^1^H NMR spectra regions of HMOs, making identification of further HMOs difficult. This was partially accounted for by using statistical total correlation spectroscopy, but implementation of more sensitive analytical techniques such as mass spectrometry may be better suited for this task. Finally, we did not stratify the *in vitro* results according to maternal GBS colonisation status. The focus of the functional assay was to assess HMO activity on GBS *in vitro* and a sample size of 40 (12 colonised women) would be too small to infer results. In subsequent studies, we would seek to assess a larger cohort of colonised women expressing each of the HMOs of interest. Fuelled by the increasing concerns about the effect of antibiotics on the infant microbiome as well as driving antimicrobial resistance, it is increasingly important that alternative methods of preventing maternal and infant colonisation with GBS are identified.

We have demonstrated *in vivo* and *in vitro* that specific HMOs associated with the Lewis and Secretor phenotype can inhibit GBS growth. Establishing whether specific HMOs are an effective adjunct to prevent colonisation in the pregnant woman and thus transfer of GBS peri-partum to her infant would be an important advance. In addition, we suggest that Lewis and Secretor phenotypes offer potential as stratification variables in clinical trials.

Mothers undergo blood tests in pregnancy to determine blood group. If those mothers who are Lewis negative are at increased risk of GBS colonisation, this simple blood test could be used to identify this group and enable supplementation throughout pregnancy. By reducing the burden of maternal, and therefore infant, colonisation, HMOs may be a low cost, non-invasive first step in reducing the burden of neonatal GBS disease globally.

## Materials and Methods

This study was nested in a larger study in The Gambia aimed to determine the association between GBS colonisation and maternally derived antibody. Methods for recruitment, sample collection and microbiological investigation have been previously described.^6^

Maternal GBS status was obtained for mothers at delivery, while infants' GBS status was determined at delivery, as well as at postnatal day 6 and 60–89 days. Infants were exclusively breast fed throughout the study period. Women were excluded from the study if they had used antibiotics within 4 weeks of labour. None of the infants were exposed to antibiotics during the study period.

One hundred and eighty-three mothers and their infants were included in the study. One hundred and nine colostrum samples, 61 breast milk samples from day 6 postpartum and 63 breast milk samples collected at 3 months postpartum were analysed. Differences in sample size between time points is due to the fact that some women were unable to donate a breast milk sample at each time point.

### Sample size

Based on results from the original study where 33.7% of women were GBS colonised and 52% of mothers transferred GBS to their infant (17.5% infants colonised) and Secretor status of 70%,^28^ a sample size of 180 women would be required to give 126 Secretor-positive women. One hundred and eighty mothers would allow at least 10 mothers in each of the 4 milk groups and give 80% power to detect a correlation of ⩾70% between HMOs and colonisation.

### Research ethics

This project was approved by The Gambia Government/MRC Joint ethics committee (Ref SCC 1350v3). Prior to enrolment, participants who met the inclusion criteria gave either written consent or, if illiterate, a thumb print, witnessed by an impartial witness.

### Colostrum and breast milk sample preparation

Mothers were provided with soap and asked to wash their hands and wipe their breasts with alcohol wipes before hand-expressing a colostrum/milk sample within the first 12 h after birth, at day 6 and between days 60 and 89. From each breast 2–3 ml of colostrum and 4–5 ml of breast milk was collected before mothers fed their infants. Milk was collected in sterile containers and immediately refrigerated at 4 °C. Samples were centrifuged at 3000 *g* for 30 min to remove lipids. Samples were frozen within 4 h of collection at −70 °C. A modified Folch extraction^30^ previously validated for use with breast milk samples was used to further remove lipids and protein from samples.^31, 32^ Finally, samples were transferred to 5 mm NMR capillary tubes for analysis. Quality control samples were prepared by pooling 50 μl of each sample and prepared in the same manner as the study samples.

### ^1^H NMR spectroscopic analysis

A Bruker 600MHz spectrometer (Coventry, UK) was used to acquire spectral data. Each sample underwent a standard 1D pulse sequence to achieve water suppression using acquisition pulse sequence using the first increment of the NOE parameters defined in Dona *et al.*^33^ and a Carr–Purcell–Meiboom–Gill pulse sequence^33^ at a probe temperature of 300 and 310 K, respectively at the Clinical Phenome Centre at Imperial College. Spectra were acquired using 4 dummy scans and 32 scans.

### Preprocessing of spectral data

Spectral data were imported into MATLAB (Mathworks, Natick, MA, USA), referencing the chemical shifts (*δ*) to glucose (α anomeric, *δ* 5.24). Regions containing the water and TSP resonance were removed from the spectra. Peak alignment was performed using recursive segment-wise peak alignment,^34^ and data were normalised using probabilistic quotient normalisation (median fold change normalisation).^35^

### Functional assay for GBS growth in breast milk

Breast milk from 40 mothers with different HMO profiles, as determined from the ^1^H NMR breast milk profiles, were selected and cultured with GBS to assess the effects of HMOs on the growth of GBS *in vitro*. Frozen stock of GBS STIII bacteria (COH-1, kind donation from Carol Baker) was defrosted and grown in Todd Hewitt Broth made selective with colistin and naladixic acid until an OD_600mm_ of 1 (1 × 10^7^ bacteria) was reached. In all, 100 μl of GBS was pipetted into a 96-well plate containing 10 μl of breast milk, and 10 μl of this preparation was plated directly onto Colombia agar (T0 sample). The remainder was incubated overnight prior to streaking onto Colombia agar. The following day, CFU were manually counted from the T0 samples and the overnight plated samples (T24). Each assay was performed in duplicate and the mean CFU ml^−1^ was used for analysis.

### Statistical analysis

Data were imported into SIMCA 14.1 (Umetrics, Malmo, Sweden) for multivariate statistical analysis using principal component analysis and OPLS. The logged bacterial abundances were used as the *Y* variable for the OPLS analysis. Pareto scaling was applied in order to scale the data. Robustness of the models was assessed by leaving every seventh sample out and back predicting into the model, and by permutation testing over 100 iterations where sample class labels are scrambled and the predictivity of model compared with the correctly classified model in order to establish the likelihood of obtaining the same statistical values by chance. Metabolites were identified based on their *δ* and peak splitting, comparing these with the literature and the Human Metabolome Database (http://www.hmdb.ca/). Statistical total correlation spectroscopy was used to aid in the assignment of the HMOs.^36^

Mothers were grouped into five milk groups based on their HMO profile, according to groupings described in Pratico *et al.*,^37^ plus an additional two milk groups detected in this study. Maternal Secretor status (Se) (mothers possessing an active FUT2 gene) was determined based on the presence or absence of peaks corresponding to 2′-FL and other structurally similar HMOs (containing an α1–2 linkage of fucose to the Galb(a1–3)GlcNAc unit of the oligosaccharide chain) in the ^1^H NMR spectra (*δ* 1.22–1.25). Maternal Lewis status (Le) (mothers possessing an active FUT3 gene) was determined based on the presence or absence of peaks corresponding to LNDFHI and LNDFHII in the ^1^H NMR spectra (*δ* 5.02–5.04). Mothers' breast milk containing α1,3-fucosylated oligosaccharides was determined by the presence or absence of peaks at *δ* 1.14–1.20 (Supplementary Table S4).

Breast milk from milk group 1, Secretor positive/Lewis positive (Se+/Le+), contained all classes of fucosylated oligosaccharides, breast milk from milk group 2 (Se−/Le+) did not contain α1,2-fucosylated structures (2′-fucosyllactose, lactodifucotetraose, lacto-*N*-fucopentaose I and LNDFHI), while individuals in milk group 3 (Se+/Le−) contained α1,2- and α1,3-fucosylated oligosaccharides but not lacto-*N*-fucopentaose II, LNDFHI and LNDFHII, characterised by α1,4-fucose linkages. Breast milk from mothers in breast milk group 4 did not contain either α1,2-fucosylated oligosaccharides or α1,4-fucosylated oligosaccharides (Se−/Le−). In addition to these groups, a final group was assigned, milk group 5, where mothers did not produce any fucosylated HMOs at all, including 3′-FL.

For the functional assay for GBS growth in breast milk, the T24 concentration was subtracted from the T0 concentration to give a reduction in CFU ml^−1^ for each sample.

GBS abundance showed skewed distributions, therefore log transformations were performed. To determine whether any associations existed between HMO profiles and GBS colonisation, two-way Pearson correlations were performed on logged bacterial abundances, correlated against the intensity value calculated from peak height at the apex of peaks at *δ*=1.15, 1.18, 1.23, 1.27, 1.29, 5.03, 5.13, 5.16, 5.27 and 5.40, corresponding to multiple HMOs.^38^

Chi-square tests were performed to determine whether any association between maternal Secretor (Se) or Lewis status (Le) and maternal and infant GBS colonisation occurred.

Univariate statistical analyses were completed using SPSS version 22 (IBM, Armonk, NY, USA) and Stata V12 (Statacorp, College Station, TX, USA). *P*-values <0.05 were considered significant. Individuals in milk group 5 were not included in the analysis as there were only two mothers in this group.

## Figures and Tables

**Table 1:** Chi-square analysis of the association between maternal Lewis antigen status and GBS colonisation at delivery

n=*109*	*Maternal Le+*	*Maternal Le−*	*Chi square*
GBS+mother	26/74 (35.1)	23/35 (65.7)	
GBS−mother	*48/74 (64.9)*	12/35 (34.3)	***X***^**2**^**=12.50;** ***P*<0.001**
GBS+infant birth	14/74 (18.9)	11/35 (31.4)	
GBS−infant birth	60/74 (81.1)	24/35 (68.6)	***X***^**2**^**=4.88;** ***P***=**0.03**
GBS+infant 60–89	14/74 (18.9)	5/35 (14.3)	
GBS−infant birth 60–89	60/74 (81.1)	30/35 (85.7)	*X*^2^=0.24; *P*=0.63

Abbreviation: GBS, Group B Streptococcus. Bold entries indicate significant values, defined as *P*<0.05.

**Table 2:** Chi-square analysis of the association between maternal HMO Secretor status and GBS colonisation

n=*109*	*Maternal Se+*	*Maternal Se−*	*Chi square*
GBS+mother	36/76 (47.4)	13/33 (39.4)	
GBS−mother	*40/76* *(52.6)*	20/33 (60.6)	*X*^2^=1.41
GBS+infant birth	20/76 (26.3)	5/33 (15.2)	
GBS−infant birth	56/76 (73.7)	28/33 (84.8)	*X*^2^=0.73
GBS+infant 60–89	12/76 (15.8)	7/33 (21.2)	
GBS−infant birth 60–89	64/76 (84.2)	26/33 (78.8)	*X*^2^=0.00

Abbreviations: GBS, Group B Streptococcus; HMO, human milk oligosaccharide.

**Table 3:** Number of mothers and infants colonised in each milk group

	*Milk group 1 Se+/Le+*	*Milk group 2 Se−/Le+*	*Milk group 3 Se+/Le−*	*Milk group 4 Se−/Le−*	*Milk group 5 Se−/Le−+no 3′-FL*	*Total*
Number	84 (46%)	35 (19%)	42 (23%)	20 (11%)	2 (1%)	All Mothers 183
Mother colonised	18/52^a^ (34%)	8/22 (36%)	18/24 (75%)	5/9 (55%)	0/2 (0%)	Colostrum 109
Infant colonised, birth	12/52 (23%)	2/22 (9%)	8/24 (33%)	3/9 (33%)	0/2 (0%)	
Infant colonised, days 60–89	8/52 (15%)	6/22 (27%)	4/24 (16%)	1/9 (11%)	0/2 (0%)	

a The denominator refers to the total number of mothers in each milk group.

**Table 4:** Chi-square analysis of the association between HMO production and GBS colonisation

	*Milk group 1 Se+/Le+*	*Milk group 2 Se−/Le+*	*Milk group 3 Se+/Le−*	*Milk group 4 Se−/Le−*
Maternal colonisation (2, *n*=183)	***X***^**2**^**=5.43,** ***P*****=0.02**	*X*^2^=2.29, *P*=0.13	***X***^**2**^**=16.57**, ***P*****<0.001**	*X*^2^=0.36, *P*=0.55
Infant colonisation, birth (2, *n*=183)	*X*^2^=1.98, *P*=0.16	*X*^2^=1.47, *P*=0.22	***X***^**2**^**=6.82,** ***P*****=0.01**	*X*^2^=0.28, *P*=0.56
Infant colonisation, days 60–89 (2, *n*=183)	*X*^2^=0.44, *P*=0.51	*X*^2^=2.03, *P*=0.15	*X*^2^=0.59, *P*=0.44	*X*^2^=2.43, *P*=0.12

Abbreviations: GBS, Group B Streptococcus; HMO, human milk oligosaccharide.Bold entries indicate significant values, defined as *P*<0.05.

**Table 5:** Two-way Pearson correlation of changes in GBS CFU ml^−1^ against the highest intensity peaks (concentration) of HMOs at specific p.p.m.s.

Intensity at δ	1.15	1.18	1.23	1.27	1.29	5.03	5.13	5.16	5.27	5.40
*Difference between CFU ml^−1^ at T0 and T24*										
Pearson correlation	−0.1	−0.12	0.09	−0.32	−0.46	−0.41	<−0.01	−0.45	−0.3	−0.13
Significance	0.54	0.47	0.57	**0.04**	**0.003**	**0.009**	0.99	**0.004**	0.06	0.42
*N*	40	40	40	40	40	40	40	40	40	40

Abbreviations: CFU, colony-forming units; GBS, Group B Streptococcus; HMO, human milk oligosaccharide. Bold entries indicate significant values, defined as *P*<0.05.

Two-tailed test.
