# Supplementary material for: Role of human milk oligosaccharides in Group B Streptococcus colonisation
Source: Clin Transl Immunology. 2016 Aug 26;5(8):e99–. doi: 10.1038/cti.2016.43 (PMC5007626; doi:10.1038/cti.2016.43)
Supplement: Supplementary Information [file cti201643x1.doc]

**Supplementary information**

**
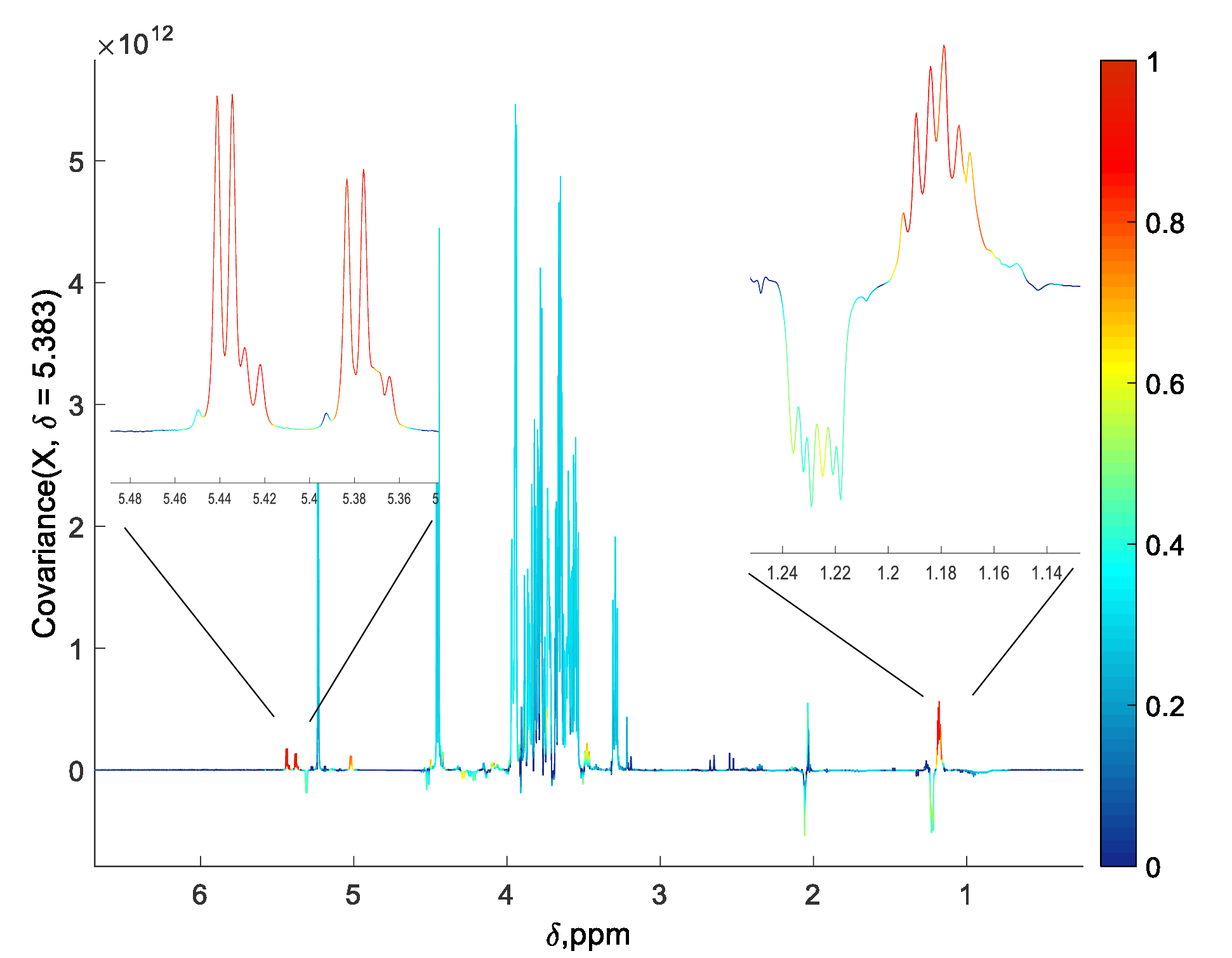
Figure 1:** STOCSY driver peak at δ 5.38, present in the structure of 3'-fucosyllactose. The resonances at δ 1.19 corresponds to H-6 in Fuc(α1-3)Glc, the peaks at δ 5.39 correspond to H-1 Fuc(α1-3)αGlc, and the peaks at δ 5.44 corresponds to H-1 Fuc(α1-3)βGlc.

**
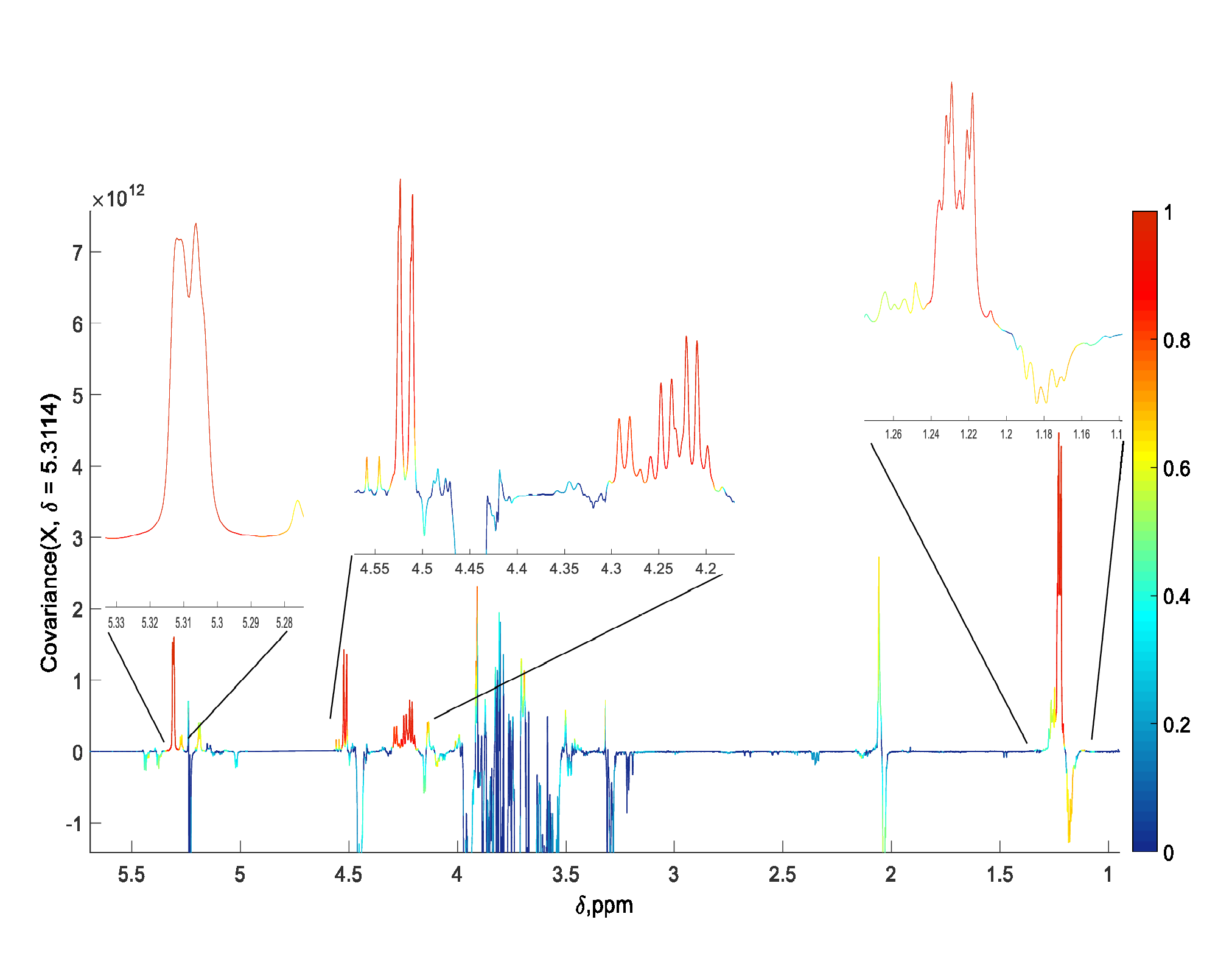
Figure 2:** STOCSY, driver peak at δ 5.31 present in the structure of 2'-fucosyllactose. The resonance at δ 1.24 corresponds to H-6 Fuc(α1-2)Glc, H-5 Fuc(α1-2) is found at δ 4.23, the peak at δ 4.53 corresponds to H-1 Gal(β1-4), and the peak at δ 5.32 corresponds to H-1 Fuc(α1-2)Gal.

**
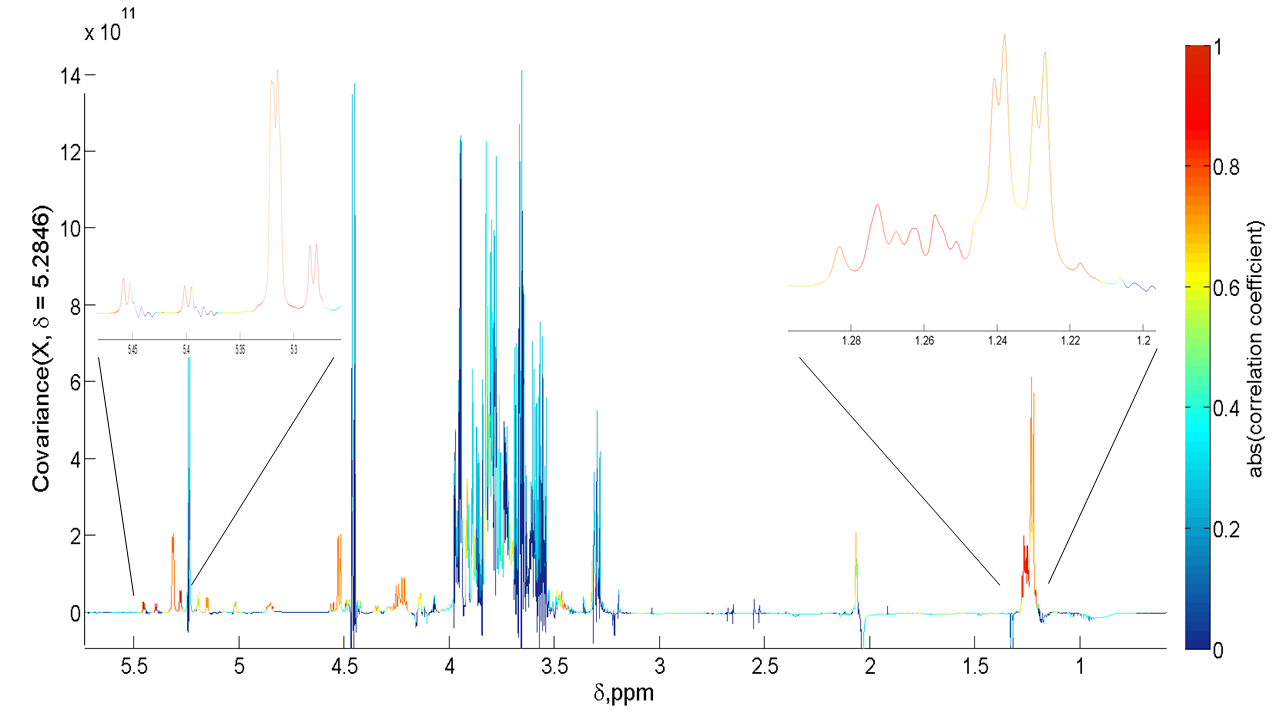
Figure 3:** STOCSY, driver peak at δ 5.28, present in lactodifucotetraose (LDFT). The resonance at δ 1.28 corresponds to H-1 Fuc(α1-2)Gal. The peak at δ 5.28 corresponds to H-1 Fuc(α1-2)Gal. The peak at δ 5.40 corresponds to H-1 Fuc(α1-3)aGlc, whilst the peak at δ 5.45 corresponds to H-1 Fuc(a1-3)βGlc.

**
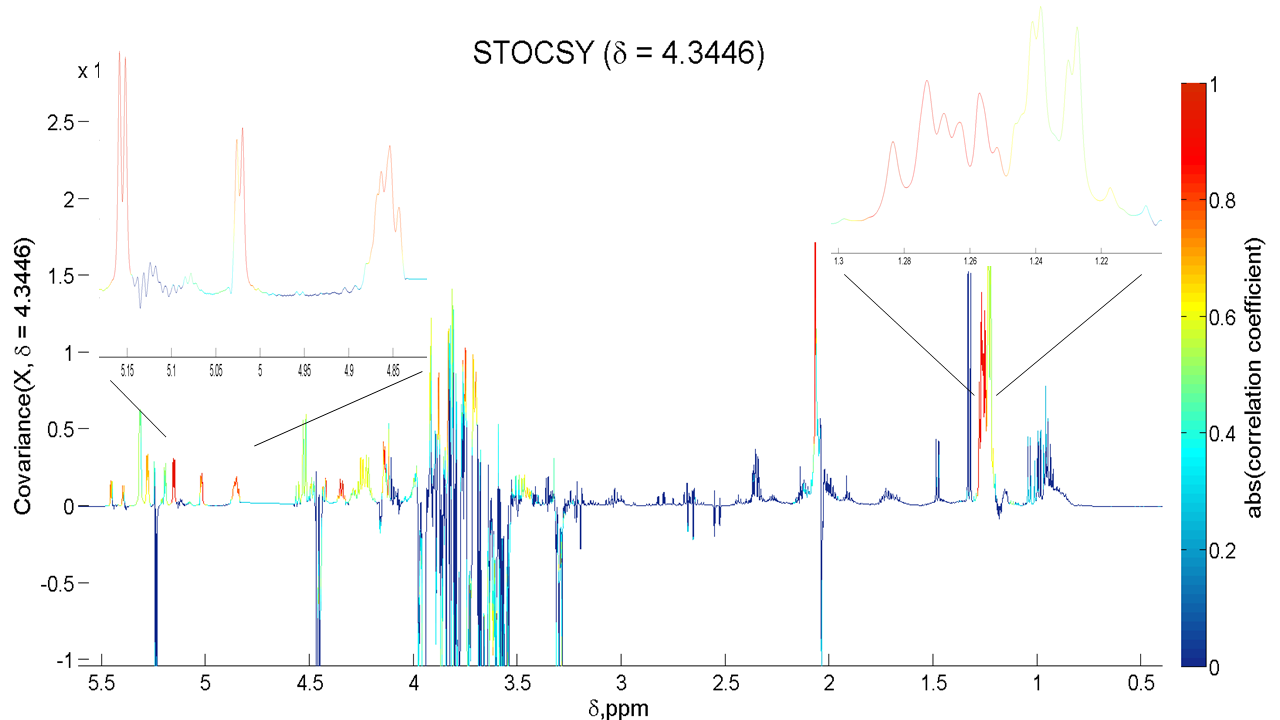
**

**Figure 4:** STOCSY, driver peak at δ 4.34, present in lacto-N-difucohexsaose I (LNDFHI) and similar branched HMO.


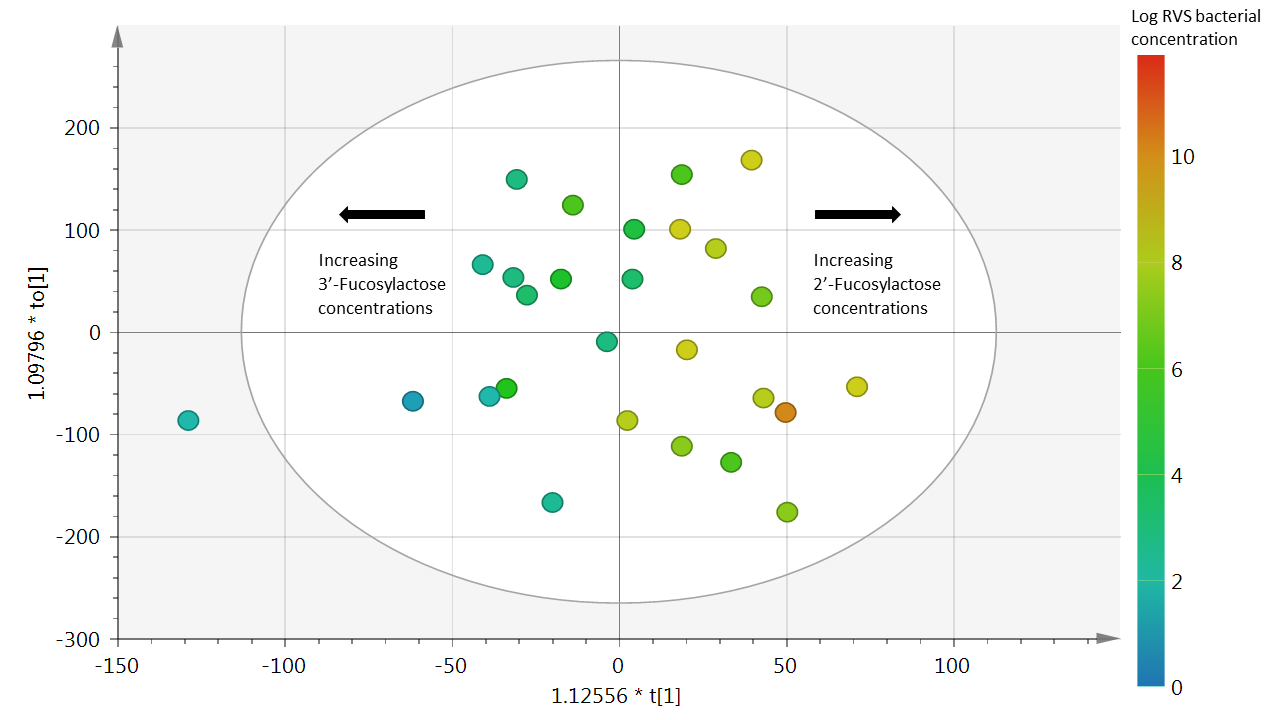
**Figure 5:** OPLS of log bacterial concentration of infant rectal swab at visit 1 against the breast milk profile, R^2^X=0.913, R^2^Y= 0.359, Q^2^=0.17. Pareto scaling.

Increasing 3’-Fucosylactose concentrations

Increasing 2’-Fucosylactose concentrations

**Figure 6:** OPLS of log bacterial concentration of breast milk at visit 1 against the breast milk profile, R^2^X=0.941, R^2^Y= 0.672, Q^2^=0.497. Pareto scaling.

**Table 1:** Two way Pearson correlation of logged bacterial abundances at visit one against the highest intensity peaks of HMO at specific ppms, two tailed test. ^*^Significant at the <0.05 level ^**^Significant at the 0.01 level.

| **Intensity at ppm** | | **1.152** | **1.18** | **1.23** | **1.266** | **1.285** | **5.025** | **5.13** | **5.155** | **5.274** | **5.403** |
| --- | --- | --- | --- | --- | --- | --- | --- | --- | --- | --- | --- |
| Log of Recto-vaginal swab | Pearson Correlation | -0.114 | -0.059 | 0.019 | 0.035 | 0.071 | -0.003 | -0.090 | 0.027 | -0.004 | 0.012 |
|  | Significance | 0.449 | 0.695 | 0.901 | 0.819 | 0.642 | 0.982 | 0.553 | 0.859 | 0.977 | 0.935 |
|  | N | 46 | 46 | 46 | 46 | 46 | 46 | 46 | 46 | 46 | 46 |
| Log Nasopharyngeal swab | Pearson Correlation | -0.173 | -0.323 | 0.371 | 0.276 | 0.319 | 0.058 | -0.230 | 0.244 | 0.192 | 0.308 |
|  | Significance | 0.418 | 0.123 | 0.074 | 0.192 | 0.129 | 0.788 | 0.280 | 0.251 | 0.368 | 0.143 |
|  | N | 24 | 24 | 24 | 24 | 24 | 24 | 24 | 24 | 24 | 24 |
| Log Rectal swab | Pearson Correlation | -0.313 | -0.540^**^ | 0.447^*^ | -0.036 | -0.178 | -0.352 | -0.356 | -0.284 | -0.005 | -0.140 |
|  | Significance | 0.112 | 0.004 | 0.019 | 0.857 | 0.374 | 0.071 | 0.068 | 0.150 | 0.979 | 0.488 |
|  | N | 27 | 27 | 27 | 27 | 27 | 27 | 27 | 27 | 27 | 27 |
| Log Breast milk | Pearson Correlation | -0.119 | -0.664^*^ | 0.593 | 0.416 | 0.119 | -0.064 | -0.348 | 0.116 | 0.518 | 0.179 |
|  | Significance | 0.743 | 0.036 | 0.071 | 0.232 | 0.743 | 0.861 | 0.325 | 0.749 | 0.125 | 0.621 |
|  | N | 10 | 10 | 10 | 10 | 10 | 10 | 10 | 10 | 10 | 10 |

**Table 2:** Two way Pearson correlation of logged bacterial abundances at visit two against the highest intensity peaks of HMO at specific ppms, two tailed test. ^*^Significant at the <0.05 level ^**^Significant at the 0.01 level.

| **Intensity at ppm** | | **1.152** | **1.18** | **1.23** | **1.266** | **1.285** | **5.025** | **5.13** | **5.155** | **5.274** | **5.403** |
| --- | --- | --- | --- | --- | --- | --- | --- | --- | --- | --- | --- |
| Log of Recto-vaginal swab | Pearson Correlation | -0.055 | -0.183 | 0.174 | -0.374 | -0.452 | -0.639^*^ | -0.146 | -0.366 | -0.205 | 0.013 |
|  | Significance | 0.857 | 0.550 | 0.569 | 0.208 | 0.121 | 0.019 | 0.633 | 0.219 | 0.502 | 0.966 |
|  | N | 13 | 13 | 13 | 13 | 13 | 13 | 13 | 13 | 13 | 13 |
| Log Nasopharyngeal swab | Pearson Correlation | -0.271 | 0.393 | -0.543 | -0.400 | -0.304 | -0.384 | -0.035 | -0.500 | -0.210 | -0.350 |
|  | Significance | 0.604 | 0.441 | 0.265 | 0.432 | 0.558 | 0.452 | 0.948 | 0.313 | 0.690 | 0.496 |
|  | N | 6 | 6 | 6 | 6 | 6 | 6 | 6 | 6 | 6 | 6 |
| Log Rectal swab | Pearson Correlation | 0.113 | 0.184 | -0.337 | 0.122 | 0.107 | 0.159 | 0.360 | 0.128 | 0.201 | -0.058 |
|  | Significance | 0.740 | 0.588 | 0.310 | 0.720 | 0.754 | 0.641 | 0.276 | 0.707 | 0.553 | 0.865 |
|  | N | 11 | 11 | 11 | 11 | 11 | 11 | 11 | 11 | 11 | 11 |
| Log Breast milk | Pearson Correlation | -0.170 | -0.191 | -0.253 | -0.396 | -0.023 | 0.272 | -0.229 | -0.256 | -0.617 | -0.737 |
|  | Significance | 0.715 | 0.682 | 0.584 | 0.379 | 0.962 | 0.554 | 0.622 | 0.580 | 0.140 | 0.059 |
|  | N | 7 | 7 | 7 | 7 | 7 | 7 | 7 | 7 | 7 | 7 |

**Table 3:** Two way Pearson correlation of logged bacterial abundances at visit three against the highest intensity peaks of HMO at specific ppms, two tailed test. ^*^ Significant at the <0.05 level ^**^Significant at the 0.01 level.

| **Intensity at ppm** | | **1.152** | **1.18** | **1.23** | **1.266** | **1.285** | **5.025** | **5.13** | **5.155** | **5.274** | **5.403** |
| --- | --- | --- | --- | --- | --- | --- | --- | --- | --- | --- | --- |
| Log of Recto-vaginal swab | Pearson Correlation | -0.184 | -0.112 | -0.205 | -0.060 | -0.076 | 0.077 | -0.232 | 0.009 | -0.047 | -0.332 |
|  | Significance | 0.330 | 0.556 | 0.276 | 0.754 | 0.689 | 0.687 | 0.217 | 0.962 | 0.803 | 0.073 |
|  | N | 30 | 30 | 30 | 30 | 30 | 30 | 30 | 30 | 30 | 30 |
| Log Nasopharyngeal swab | Pearson Correlation | 0.307 | -0.004 | -0.139 | -0.198 | -0.371 | -0.193 | 0.249 | -0.199 | -0.029 | -0.387 |
|  | Significance | 0.503 | 0.993 | 0.766 | 0.670 | 0.412 | 0.678 | 0.591 | 0.669 | 0.950 | 0.391 |
|  | N | 7 | 7 | 7 | 7 | 7 | 7 | 7 | 7 | 7 | 7 |
| Log Rectal swab | Pearson Correlation | 0.066 | 0.355 | -0.131 | 0.207 | 0.204 | 0.056 | 0.012 | 0.155 | 0.069 | 0.385 |
|  | Significance | 0.794 | 0.148 | 0.605 | 0.410 | 0.418 | 0.826 | 0.962 | 0.540 | 0.785 | 0.115 |
|  | N | 18 | 18 | 18 | 18 | 18 | 18 | 18 | 18 | 18 | 18 |
| Log Breast milk | Pearson Correlation | -0.197 | 0.030 | -0.212 | -0.534 | -0.269 | -0.218 | -0.470 | -0.675 | 0.077 | -0.231 |
|  | Significance | 0.672 | 0.949 | 0.648 | 0.217 | 0.560 | 0.638 | 0.287 | 0.096 | 0.870 | 0.618 |
|  | N | 7 | 7 | 7 | 7 | 7 | 7 | 7 | 7 | 7 | 7 |

Table 4: Chemical shifts of fucosylated oligosaccharides and their associated milk groups.

| **Chemical Shift (δ)** | **Associated structure(s)** | **Associated Oligosaccharide(s)** | **Group 1**  **Se^+^/Le^+^** | **Group 2**  **Se^-^/Le^+^** | **Group 3**  **Se^+^/Le^-^** | **Group 4**  **Se^-^/Le^-^** | **Group 5**  **Se^-^/Le^-^ plus no 3’ oligosaccharides** |
| --- | --- | --- | --- | --- | --- | --- | --- |
| 1.24 | H-6 Fuc(α1-2)Glc  H-3 Fuc(α1-2)Gal | 2’-fucosyllactose  LNFPI and branched | ✓ | × | ✓ | × | × |
| 5.03 | H-1 Fuc(α1-4)GlcNAc | LNDFHI and branched | ✓ | ✓ | × | × | × |
| 1.19 | H-6 Fuc(α1-3)Glc | 3’-fucosyllactose | ✓ | ✓ | ✓ | ✓ | × |
